# Serial enrichment incubation technique (SEIT) for rapid isolation of plant growth-promoting bacteria: Saving researcher time and lab resources

**DOI:** 10.1016/j.mex.2025.103677

**Published:** 2025-10-14

**Authors:** Hemant Singh Maheshwari, Deepika Parmar, Aakash Gour, Akanksha Patel, Sanjeev Kumar, Laxman Singh Rajput, Nataraj Vennampally, Jeberlin Prabina Bright, Rakesh Kumar Singh, Mahaveer Prasad Sharma, Kunwar Harendra Singh

**Affiliations:** a ICAR-National Soybean Research Institute, Indore, India; b University of Groningen, The Netherlands; c ICAR-Central Arid Zone Research Institute, Jodhpur, Rajasthan, India; d Tamil Nadu Agricultural University, Coimbatore, India; e Rajmata Vijayaraje Scindia Krishi Vishwavidyalaya, Gwalior, Madhya Pradesh, India

**Keywords:** Serial enrichment incubation technique (SEIT), Siderophore producer, Phosphorus solubilizer, Potassium solubilizer, Zinc solubilizer, Calcium solubilizer, Dye

## Abstract

Traditional methods of Plant growth-promoting bacteria (PGPB) isolation involve serial dilution and spreading on common or specific media, which leads to more than 10 bacteria per sample, and screening of these bacteria for a desired potential bacterium takes much time (4-6 months) and lab resources. The current improved protocol, termed Serial Enrichment Incubation Technique (SEIT), involves incubating the sample in a specific nutrient medium for 5 days, then transferring it into a fresh medium twice for five days each, and finally serially diluting the final tube and spreading the suspension onto Petriplates containing specific media. This protocol gives the desired nutrient-transforming bacteria compared to traditional published protocols within 20 days. Isolation of the siderophore-producing bacteria (SPB) by incubating in an iron-free succinate medium allows the elimination of non-siderophore-producers, and these isolates are also potential candidates for biocontrol against phytopathogens. Soil nutrient solubilizers can be isolated rapidly using SEIT.

• SPB can be isolated by incubating in iron-free succinate media and could be used for iron nutrition and biocontrol against phytopathogens.

• Efficient PGPB could be isolated through SEIT in less than 20 days.

• Slow-growing and less populated PGPB can be isolated, and its inoculation would increase soil microbial diversity.

## Graphical abstract

**Figure:**
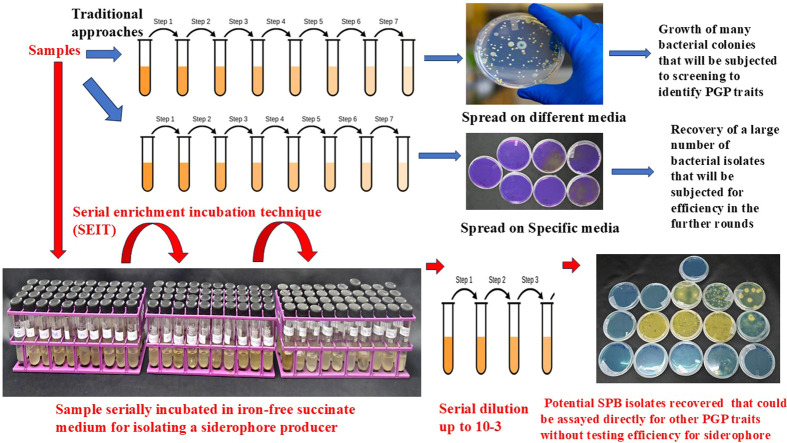

## Specification table

**Table:** 

**Subject area**	
**More specific subject area**	Soil Microbiology
**Name of your protocol**	Serial Enrichment Incubation Technique (SEIT)
**Reagents/tools**	Mentioned in each section
**Experimental design**	Serially incubating the samples in specific media with altered nutrients/chemicals that allow the growth of desirable plant growth-promoting bacteria by three consecutive enrichments.
**Trial registration**	Not applicable
**Ethics**	Not applicable
**Value of the Protocol**	The serial enrichment incubation technique (SETI) facilitates the isolation of potential soil nutrient-transforming bacteria in a shorter period (20 days) than the traditional screening method. Plant growth-promoting bacteria (PGPB), such as solubilizers of phosphorus, potassium, zinc, and calcium, could be isolated by serially incubating the sample in their respective insoluble source, allowing growth of only the efficient nutrient transformers through three incubations. Potential nutrient-solubilizing bacteria in lower abundance and slow-growing strains could be isolated, which is impossible with the traditional method. This would help to increase the population through soil inoculation, which in turn favours microbial diversity. Potential siderophore-producing bacteria with biocontrol traits (plant health promoters) could be isolated by incubating them in an iron-free succinate medium compared to the traditional method of serial diluting and directly spreading them onto nutrient agar or CAS agar plates.

## Background

Plant growth-promoting bacteria (PGPB) utilize various direct and indirect mechanisms for plant growth promotion. These PGPB are therefore termed as solubilizers of phosphorus, potassium, zinc, calcium, silicon, siderophore producers, and sulfur oxidizers [1,2]. Previous studies showed that many researchers isolated these PGPB through serial dilution of the sample followed by spreading into nutrient agar medium or any specific media, and then bacteria were picked from the plates, the zones, or the discolored portion in the Petri plates [[3], [4], [5]]. However, these two traditional methods allow the growth of other bacteria due to the presence of glucose or any nutrient in the media composition. Furthermore, these two methods lead to the growth of approximately >10 bacteria on plates. Then, each isolate was purified and tested for its plant growth promotion activities. Currently, it takes lots of time, consumes lots of chemicals, Petri plates, and presents difficulties in maintaining and screening a large number of bacterial isolates for potential PGPB [[3], [4], [5]]. Further, less abundant and slow growers with higher solubilization efficiency failed to grow with traditional methods, as fast growers outnumbered the Petri plates. Additionally, the growth of unwanted bacteria may become a common contaminant in the microbiology labs.

### Description of protocol

In the modified protocol, we provide a selection pressure by serially incubating the samples in an insoluble source medium through enrichment. This results in the selective growth of the potential PGPB. In the modified techniques of isolation of PGPB, we took 0.1 g of crushed plant or soil sample, or 0.1 ml in case of liquid samples. We transferred it into 10 ml specific media containing an insoluble nutrient source with a pH of 8.00. Insoluble sources of phosphorus (tricalcium phosphate), calcium (calcium carbonate or calcium phosphate), potassium (mica or potassium aluminosilicate), and zinc (zinc carbonate) were used. Nystatin antibiotic 50 mg ml^−1^ media was added to check the fungal contamination. Further, dyes such as bromophenol blue, bromothymol blue, and bromocresol purple were added as pH indicators, which helped visual identification of solubilizers due to a decrease in the pH of the medium through the production of organic acids. Then, from positive discolored tubes, 100 µl of the suspension was further transferred to a fresh medium till the discolouration of the medium occurred. Discolouration of the media was due to changes in pH brought by the desired bacteria in the samples due to organic acid production that aids in solubilizing the insoluble form of nutrients. Therefore, this step can be used to screen samples rapidly, and non-discoloured samples can be eliminated.

A similar series of transfers followed by incubation was repeated twice. From the final tubes, the suspension was serially diluted to 10^−3^, and 100 µl of the 10^−3^ dilution was spread uniformly onto the respective agar amended media with dye. These petriplates were kept for 5 days at 30 ±2°C till the colour change was visible and retained for 24 hours. Only stable colour changed plates with a clearing zone around the bacterial colony were further purified in the nutrient agar for further work. In this process, only a few (1-2) positive isolates with desired traits were grown on the Petriplates, making isolation and screening easier in one series of processes. The whole process was completed in less than 20 days, and the desired bacteria were isolated from environmental samples. Traditional isolation methods and further screening for traits like efficiency, sustained activity, and high solubilization index would take 4-6 months and consume lots of reagents and Petri plates to screen samples or bacteria before getting a potential one. Additionally, in the proposed method, the time taken for the tubes to change colour showed the efficiency of the bacteria. An early change of colour tube showed greater potential in converting insoluble to soluble form, indicating the organic acid production efficiency.

Under SEIT, media composition could be modified with the vital nutrient related to the trait, such that their food source for growth and metabolism solely depends on a particular compound. Other plant growth promotion traits of PGPB, such as phytohormone production, exopolysaccharide synthesis, and ACC-deaminase activities, can be isolated by altering the composition of minimal media with the addition of solely precursors/substrates. Further incubation would selectively allow the growth of desired bacteria with traits, and others would be eliminated. Other plant growth-promoting microorganisms, such as fungi, actinomycetes, and yeast, can be isolated using the same methodology. Differences between traditional protocols and SEIT have been described in Table 1.

**Table 1:** Difference between previously published protocols and the Serial enrichment incubation technique (SEIT).

Sr no	Parameters	Previously published protocols	SEIT
1	Methodology	Serially dilute the samples until 10^−5^and place them directly into common media, like nutrient agar or specific media.	Placing samples into selective media/compounds/precursors, as the sole source of food for the bacteria, with three successive incubations
2	Type of bacteria	It allows both bacteria of interest and non-interest	Selectively allow growth of the bacteria of choice.
3	Number of bacteria per sample	More than 10 per sample	Incubation allows a maximum of 1-2 desirable bacteria
4	Growth rate	Only fast growers appear on plates	Both fast and slow-growing bacteria can be isolated
5	An abundance of bacteria in the samples	Less chance of recovery of thinly populated bacteria	Thinly populated bacteria can be isolated.
6	Screening of a sample containing desirable bacteria	Not possible	Possible since tubes showing non-discoloration can be screened out for further incubation, saving time and resources.
7	Time	Screening required to get 1-2 potential ones for commercial application	No screening required as only a few potential ones appear on Petri plates.
8	Resources	Many resources (chemicals, time, and labour) are required to screen potential ones.	Less resources
9	Maintenance of culture	High due to the large number of recoveries of bacteria per sample.	Less maintenance
10	Time	Minimum 6 months	Quickly, as potential ones recovered within 20 days.
11	Future implication	-	Use the same idea for isolating PGP traits like ACC deaminase, exopolysaccharides synthesis, and phytohormone production. The same idea can be applied to isolating other beneficial microorganisms like fungi, actinomycetes, cyanobacteria, and yeasts with industrial importance.
12	Limitation	A large number of fast-growing bacteria were recovered. Only the culturable fraction of microorganisms is isolated.	A whole bacterial diversity is not possible. Only the culturable fraction of microorganisms is isolated.

### Equipment and consumables required

Autoclave

Laminar air flow

Bacteriological filter 0.22 µm

Nystatin (Himedia)

Sterilized needles

Conical flask- 500 ml

Culture tubes (30 ml capacity)

Micropipette and Sterilized pipette tips (1 ml)

### Protocol validation

The following section discusses the protocol validation for siderophore-producing bacteria, solubilizers of calcite, phosphorus, potassium, and zinc. Discolouration or a zone of clearing in specific media petriplates indicates the presence of desirable PGPB. The difference between the methodology and the findings of previously published protocols was discussed in Table 2.

**1. Isolation of Siderophore-producing bacteria (SPB) from extreme habitats.**

**Table 2:** Comparative protocol between traditional and Serial Enrichment Incubation Technique.

Nutrient-transforming bacteria	Serial enrichment incubation technique (SEIT)	Traditional approaches
Siderophore-producing bacteria (SPB)	Serial incubation in an iron-free succinate medium was done three times, followed by serial dilution of the final tube and spreading on the CAS plate (Fig. 1). We got seven bacteria from 15 samples based on discoloration on CAS agar plates, from blue to orange.	Serial dilution of soil samples and spreading on nutrient agar led to 33 bacteria, and then screened for individual bacteria on a CAS plate and got 17 positive for siderophore [13]. Soil samples were serially diluted and placed on Luria-Bertini agar plates, and 32 bacteria were obtained, among which 13 showed positive for siderophore upon confirmation in CAS plate [14].
Phosphorus-solubilizing bacteria (PSB)	Samples were serially incubated in NBRIP broth three times, followed by serial dilution of the final tube and spreading on the NBRIP agar plate. We got three PSB from 15 samples based on the zone of solubilization in NBRIP agar plates.	Soil samples serially diluted and plated on Luria-Bertani medium led to the isolation of 55 bacteria, and among these, two bacterial cultures were promising [15].
Calcium-dissolving bacteria	Samples were serially incubated in calcium differentiating broth three times, followed by serial dilution of the final tube and spreading on calcium differentiating agar plates. Out of 8 samples under study, three calcium-solubilizing bacteria were found.	Soil collected from the peanut field showed 65 bacteria upon serial dilution and plating on a calcium-differentiating agar plate [16]. However, these strains must be screened to get potential ones for field-level application.
Potassium-solubilizing bacteria	Samples were incubated three times in Alexandrov broth containing dye, and then the final tubes were serially diluted, and the suspension was spread on Alexandrov agar plates. Out of 8 samples under study, five bacterial isolates were potassium solubilizers.	Serial dilution of the sample and placing on Alexandrov medium led to the recovery of 24 bacteria, which were further screened to get two potential ones [17]. Similarly, 80 bacteria were isolated from the rhizosphere soil of maize and wheat through serial dilution and plating on an Aleksandrov agar plate supplemented with mica, and finally, they identified 44 KSBs. These need further screening for practical utility for field application [18].
Zinc-solubilizing bacteria	Samples are serially incubated in zinc broth containing insoluble zinc carbonate containing dye, then the final tubes are serially diluted and the suspension is spread on zinc agar plates. Two zinc-solubilizing bacteria from 8 samples.	Samples collected from rice rhizosphere, non-rhizosphere, and endophytes were serially diluted and plated in nutrient agar, isolating 88 bacterial isolates. These were further screened qualitatively, and only nine showed positive for zinc solubilization [19]. Rhizosphere soil was placed in sterile water for two hours with shaking, followed by serial dilution and plating on nutrient agar plates, which led to the isolation of 121 bacteria. These bacteria were further screened qualitatively, and only six showed the zinc solubilization potential [20].

Many previous studies showed that isolation of siderophore bacteria involves serial dilution of samples and spreading into a chromoazurol sulfonate-nutrient agar medium [6,7]. However, in the improved protocol, we used an iron-free succinate medium directly, which is used for the quantification of siderophore potential. To our knowledge, we used an iron-free succinate medium for the first time to isolate siderophore-producing bacteria. Previously, in the lab, we observed that greater turbidity in iron-free succinate medium showed greater quantities of siderophore. Therefore, we hypothesized that samples carrying SPB would grow on the iron-free succinate medium and give turbidity. In contrast, samples upon incubation lacked turbidity, indicating a lack of the desired bacteria. This approach is novel for isolating siderophore-producing bacteria, as only the siderophore producer can grow inside the iron-free succinate medium, and any non-siderophore producer may be eliminated upon prolonged incubation. Additionally, this protocol may be used for screening for biocontrol action against phytopathogens, as siderophore production is one of the mechanisms for checking the growth of phytopathogens by scavenging iron. The SEIT procedure allows isolation of siderophore-producing bacteria from both iron-rich and iron-deficient samples (Supplementary Fig. 1).

### Media preparation

**1.** Composition (g L^−1^) for iron-free succinate medium: K_2_HPO_4_- 6.0, KH_2_PO_4_-3.0, MgSO_4_.7H_2_0- 0.2, (NH_4_)_2_SO_4_- 1.0, and succinate- 4.0, and pH 7.00.
2. Chrome Azurol Sulfonate (CAS) agar plate composition: (solution A: 60.5 mg of Chromoazural sulfonate in 50 ml of distilled water, solution B: Mix 10 ml of 2 mM FeCl_3_.6H_2_0 with 10 mM HCl while stirring, and solution C: 72.9 mg of HDTMA is dissolved in 40 mL of distilled water. Solution A is poured with freshly prepared 10 ml of Solution B while stirring and mixing without forming foam. Finally, slowly adding Solution C will give a bright blue solution of 100 mL. Now, 100 ml CAS dye solution and 900 ml nutrient agar were autoclaved separately and added together while stirring in a laminar airflow before pouring into sterilized Petri plates [8].

**Procedure:**

1. Here, 15 soil and liquid water samples were collected from Chhattisgarh, India's coal and iron mines, and hot water spring areas.
2. An iron-free succinate medium was prepared, and 10 ml was dispensed into a 30 ml culture tube. Antifungal compounds, nystatin 50 µg ml^−1^ medium, were added to prevent fungal contamination during incubation of samples.
3. Solid samples 1g or liquid 1 ml were inoculated in the 10 ml culture tube for 5 days at 28 ± 2°C, and then 100 µl of the sample was transferred to a new broth aseptically, and this transfer to fresh media broth was carried out twice for incubation for 5 days.
4. Final tubes were serially diluted into 10^−1^, 10^−2,^ and 10^−3^, and from the 10^−3^ dilution sample, 100 µl of a portion was spread on a CAS-nutrient agar plate.
5. Conversion of blue to orange on CAS plates was taken as a positive for the siderophore production assay and restreaked into new plates (Fig. 1).

**Fig. 1:**
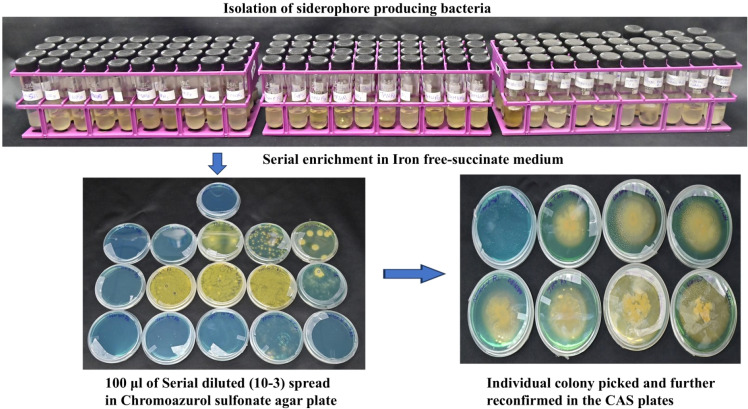
Isolation of siderophore-producing bacteria from iron mines using iron-free succinate medium.
6. The recovered bacteria were further assessed qualitatively in CAS-nutrient agar plates for validation.
7. Out of 15 samples under study, seven siderophore-producing bacteria were found as discolouration of CAS agar plates from blue to orange (Fig. 1).
2. Isolation of phosphate-solubilizing bacteria from Coal & iron mines.

· National Botanical Research Institute phosphate growth medium (NBRIP) broth for Phosphorus-solubilizing bacteria [9]; (Composition g L^−1^: D-glucose- 10.00, Ca_3_(PO_4_)_2_- 5.00, MgCl_2_.6H_2_O- 5.0, MgSO_4_.7H_2_O-0.25, KCl-0.2, (NH_2_)_2_SO_4_-0.10, and pH adjusted to 7.0. Bromophenol blue-0.01 g L^−1^ for easier identification and solidifying, 18 g L^−1^ agar-agar was added to NBRIP broth.
· In broth media, antifungal nystatin was added at 50 µg ml^−1^.

**Procedure:**

1. Coal and iron mines (1.0 g or 1 ml) samples were directly placed in a 10 ml NBRIP broth for phosphorus solubilizers.
2. After 5 days of incubation, the tube showing yellow discoloration was transferred to fresh NBRIP broth aseptically, and this step was done twice.
3. Final tubes were serially diluted into 10^−1^, 10^−2^, and 10^−3^, then spread into NBRIP agar plates for phosphate solubilizer and purified again (Fig. 2).

**Fig. 2:**
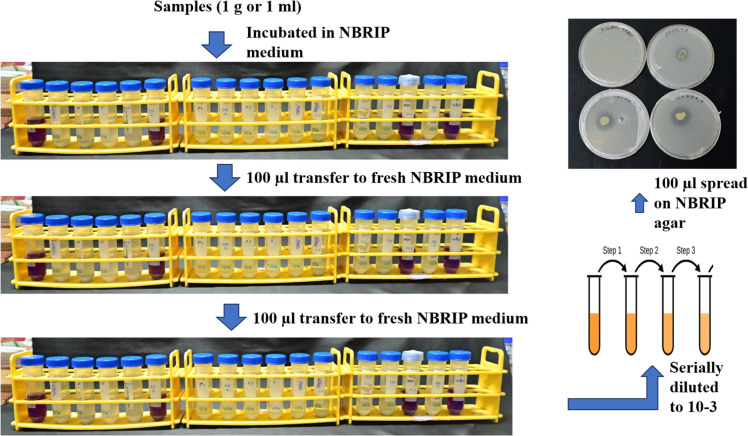
Isolation of phosphorus-solubilizing bacteria from coal & iron mines.
4. Reconfirmation of phosphorus-solubilizing PGPB was done through streaking NBRIP agar plates.
5. Out of 15 samples under study, three phosphorus-solubilizing bacteria were recovered based on the zone of solubilization in NBRIP agar plates (Fig. 2).
3. **Isolation of calcium-dissolving bacteria (CDBs) from plant samples.**

**Medium preparation:**

· Calcium differentiating broth medium for calcium solubilizer [10]: (composition g L^−1^: Glucose- 5.00, Yeast extract- 1.00, peptone- 1.00, K_2_HPO_4_- 0.4, MgSO_4_.7H_2_O- 0.01, NaCl- 5.00, (NH_4_)_2_SO_4_- 0.05, and Calcium carbonate- 5.00). Antifungal nystatin 50 µg ml ^−1^ and bromothymol blue dye- 0.01 g L^−1^ were added.
· For calcium-differentiating agar plates, 18 g L^−1^ agar-agar was added to the broth medium.

**Procedure:**

**Fig. 3:**
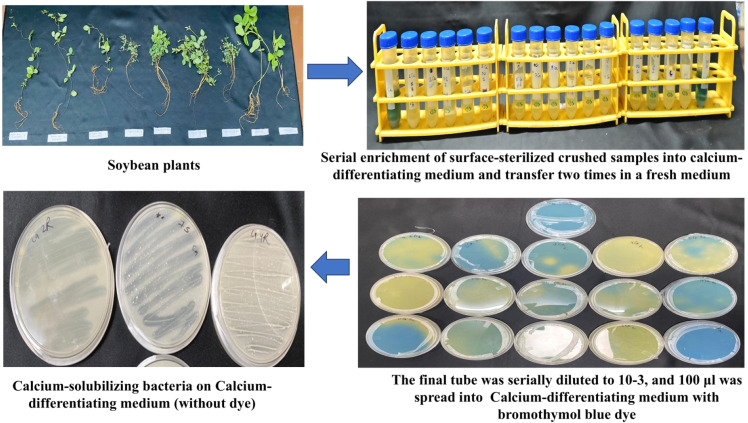
Isolation of calcium-dissolving bacteria through enrichment in calcium-differentiating medium.
**5. Isolation of potassium-solubilizing bacteria (KSBs) and zinc-solubilizing bacteria (ZSBs)**

1. One ml surface-sterilized aliquot of plant sample (previous steps) was inoculated into 10 ml calcium-differentiating medium for five days. Tubes showing yellow discoloration were serially incubated twice aseptically for 5 days each.
2. Final tubes were serially diluted until 10^−3^, and 100 µl was spread onto Calcium differentiating agar plates, and discoloured plates were taken as positive for calcium solubilization.
3. Then again, bacterial cultures were purified into new calcium-differentiating agar plates for reconfirmation. A transparent zone around the bacterial culture indicates the presence of a calcium solubilizer.
4. Out of 8 samples under study, three calcium-solubilizing bacteria were found based on clearing calcium-differentiating agar plates (Fig. 3).

### Medium preparation

Aleksandrov medium was used to isolate potassium-solubilizing bacteria. Composition of media g L^−1^ [11]: Dextrose- 5.00, MgSO_4_.7H_2_0- 0.50, FeCl_3_- 0.005, CaCO_3_-0.10, Ca_3_(PO_4_)2- 2.00, KAlSi_3_O_8_- 2.00, and bromothymol blue- 0.01.

Zinc solubilizing bacteria, composition of basal media g L^−1^ [12]: Dextrose- 10.00, (NH_4_)_2_SO_4_-1.00, KCl-0.20, K_2_HPO_4_-0.10, MgSO_4_.7H_2_0-0.10, ZnCO_3_-1.00. Additionally, bromothymol blue- 0.01 g L^−1^ was added for clear identification.

In both the medium, Nystatin was added at 50 µg ml^−1^, and for solidifying, 18 g L^−1^ agar-agar was added.

**Procedure:**

**Fig. 4:**
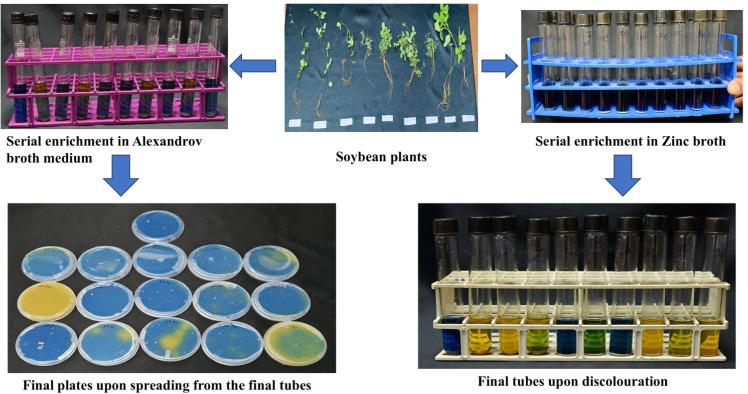
Isolation of potassium-solubilizing and zinc-solubilizing bacteria.

1. One ml surface-sterilized aliquot of plant sample (previous steps) was inoculated into 10 ml of Aleksandrov and zinc broth, incubated for five days, and transferred to a new medium twice.
2. Final tubes were serially diluted to 10^−3^, spread on respective agar plates, and purified.
3. Discolouration to yellow from blue or green indicates the presence of desirable potential bacteria.
4. Out of 8 samples under study, five potassium solubilizers and two zinc solubilizing bacteria were found to be recovered (Fig. 4)

## Limitations

This Serial enrichment incubation technique is a quicker way to get the desired specific nutrient-transforming PGPB; however, it failed to understand the whole diversity of PGPB in the given sample. Like traditional approaches, only the culturable fraction of the bacteria would be recovered from the sample, but in a rapid manner. Sometimes we failed to isolate the desired PGPB from discoloured tubes upon incubation, as non-culturable PGPB changed discoloration.

## CRediT authorship contribution statement

**Hemant Singh Maheshwari:** Conceptualization, Methodology, Resources, Writing – original draft. **Deepika Parmar:** Investigation, Writing – review & editing. **Aakash Gour:** Investigation, Writing – review & editing. **Akanksha Patel:** Investigation, Writing – review & editing. **Sanjeev Kumar:** Conceptualization, Methodology, Resources, Writing – original draft. **Laxman Singh Rajput:** Conceptualization, Methodology, Resources, Writing – original draft. **Nataraj Vennampally:** Supervision, Methodology, Writing – review & editing. **Jeberlin Prabina Bright:** Supervision, Methodology, Writing – review & editing. **Rakesh Kumar Singh:** Supervision, Methodology, Writing – review & editing. **Mahaveer Prasad Sharma:** Supervision, Methodology, Writing – review & editing. **Kunwar Harendra Singh:** Supervision, Methodology, Writing – review & editing.

## Declaration of competing interest

The authors declare that they have no known competing financial interests or personal relationships that could have appeared to influence the work reported in this paper.

## Data Availability

No data was used for the research described in the article.

## Appendix Supplementary materials

Image, application 1
