# Supplementary material for: Serial enrichment incubation technique (SEIT) for rapid isolation of plant growth-promoting bacteria: Saving researcher time and lab resources
Source: MethodsX. 2025 Oct 14;15:103677. doi: 10.1016/j.mex.2025.103677 (PMC12569831; doi:10.1016/j.mex.2025.103677)
Supplement: Supplementary file 1 [file mmc1.docx]

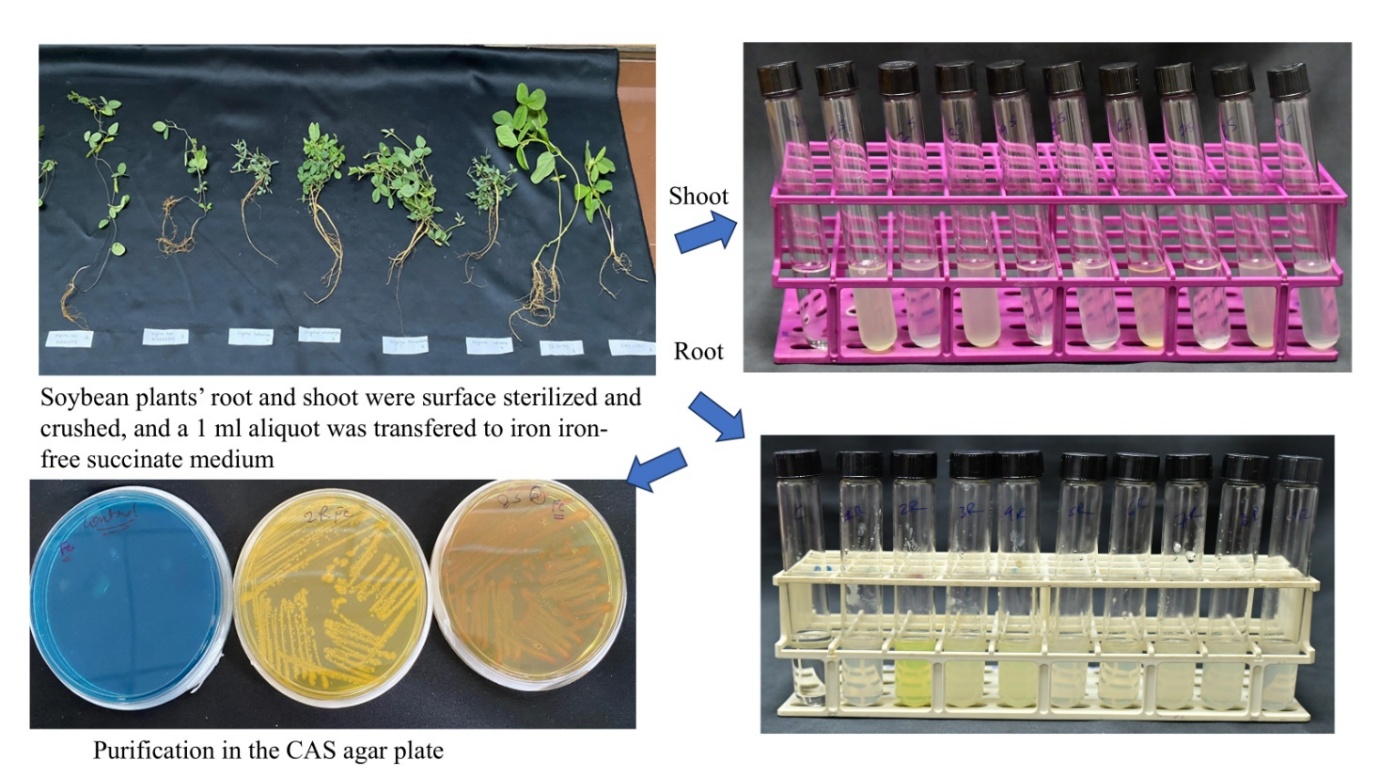


**Supplementary Fig. 1. Isolation of siderophore-producing bacteria from soybean endophytes (wild and modern cultivars) grown in vertisols (pH>8.00)**
